# Association of Demographic, Clinical, and Environmental Factors With Disease Severity in Adult Myasthenia Gravis: A Multicenter Cross‐Sectional Study

**DOI:** 10.1002/cns.71104

**Published:** 2026-08-25

**Authors:** Haoran Liu, Jingsi Wang, Wenjia Zhu, Ting Chang, Hongxi Chen, Chao Sun, Rongjing Guo, Zhe Ruan, Fangfang Bi, Jing Li, Jianwen Wang, Shu Zhang, Qi Wen, Yaye Wang, Yun Li, Nairong Xie, Yuting Jiang, Yan Lu, Li Di, Min Xu, Min Wang, Hai Chen, Xinmei Wen, Jianying Duo, Yue Huang, Huiyu Feng, Hongyu Zhou, Yuwei Da

**Affiliations:** ^1^ Department of Neurology Xuanwu Hospital, Capital Medical University Beijing China; ^2^ Department of Neurology Tangdu Hospital, The Fourth Military Medical University Xi'an China; ^3^ Department of Neurology West China Hospital, Sichuan University Chengdu China; ^4^ Department of Neurology Xiangya Hospital, Central South University Changsha China; ^5^ Department of Neurology The First Affiliated Hospital, College of Medicine, Zhejiang University Hangzhou China; ^6^ Department of Neurology The First Affiliated Hospital of Sun Yat‐Sen University Guangzhou China

**Keywords:** factor, lifestyle, myasthenia gravis, natural environment, severity, socioeconomic status

## Abstract

**Background:**

The severity of myasthenia gravis (MG) varies significantly among individuals, yet the factors underlying this heterogeneity are not fully understood. This study aimed to investigate the association between demographic, clinical, and environmental factors and the severity of MG.

**Methods:**

From February 2017 to December 2021, adult patients registered in the national MG database were enrolled in this cross‐sectional study. Baseline data on demographic, clinical, and environmental factors (including the natural environment, socioeconomic status, and lifestyle) were collected. Disease severity was classified as mild or moderate to severe using the MG Activities of Daily Living (MG‐ADL) scale and the Quantitative MG (QMG) scale. Logistic regression was used to estimate odds ratios (ORs) and 95% confidence intervals (CIs) for these associations.

**Results:**

A total of 2468 patients were included. The median MG‐ADL score was 3.0 (interquartile range [IQR] 1.0–6.0), and the median QMG score was 6.0 (IQR 3.0–9.0). Higher MG‐ADL scores were associated with age at onset ≥ 65 years (OR 1.85, 95% CI 1.32–2.58), underweight (2.03, 1.35–3.06), generalized onset (2.80, 2.26–3.48), and thymoma (1.70, 1.26–2.30). In contrast, overweight or obesity (OR 0.65, 95% CI 0.52–0.82), medium altitudes (0.64, 0.46–0.90), high educational attainment (0.68, 0.49–0.94), and high monthly household income (0.71, 0.53–0.96) were inversely associated with MG‐ADL scores. Factors associated with higher QMG scores included female sex (OR 1.75, 95% CI 1.28–2.37), age at onset ≥ 65 years (1.91, 1.35–2.71), generalized onset (2.50, 2.00–3.13), thymoma (1.97, 1.43–2.71), medium latitudes (1.53, 1.08–2.16), and high altitudes (1.65, 1.10–2.47). Lower QMG scores were correlated with overweight or obesity (OR 0.78, 95% CI 0.61–0.99), thymectomy (0.57, 0.40–0.81), medium altitudes (0.70, 0.49–0.99), and medium sunlight duration (0.67, 0.49–0.93), as well as higher concentrations of particulate matter with an aerodynamic diameter of ≤ 2.5 μm and higher household income.

**Conclusion:**

In addition to demographic and clinical factors, environmental factors may also influence MG severity. Further research is warranted to clarify these associations and their underlying mechanisms.

## Graphical abstract

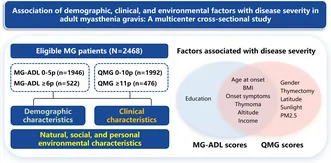

The disease severity of adult myasthenia gravis is influenced by multiple factors. Our results showed that gender, age at onset, BMI, onset symptoms, thymoma, and thymectomy were correlated with disease scores. Additionally, environmental exposures, including latitude, altitude, sunlight duration, PM2.5, education, and monthly household income, also had an impact on disease activity.

## 1 Introduction

Myasthenia gravis (MG) is an antibody‐mediated autoimmune disease (AID) characterized by fluctuating fatigability and muscle weakness [1]. Over the past few decades, advances in diagnosis and immunotherapy have improved the management of MG. However, disease severity still varies substantially across patients. Most patients achieve minimal symptom expression within 2 years of starting treatment [2] but approximately 10%–30% remain refractory to available therapies [3]. In addition, life‐threatening events continue to pose a major clinical challenge [1]. Early identification of patients at higher risk for poor outcomes is essential for timely intervention and better prognosis.

Growing evidence suggests that variations in the clinical course of MG are influenced by multiple factors. Demographic and clinical characteristics, such as age at onset, sex, pathogenic antibodies, thymic features, and immunotherapy, are widely associated with clinical outcomes [4, 5]. Few studies have evaluated the impact of environmental factors on MG, and the results are often inconclusive. In a study by Apinyawasisuk et al., smoking was found to increase the risk of generalization in ocular MG [4]. Our previous cohort study did not confirm this pattern but instead identified latitude and income as potential risk factors for generalization [6]. Additionally, a cross‐sectional study from Sweden reported that patients with university education had milder symptoms [7]. The human environment consists of natural, social, and personal domains, which play an important role in the development of various AIDs [8]. Geographical settings, socioeconomic status (SES), and lifestyle are key indicators used to evaluate environmental exposures [8, 9]. However, the measurements vary across prior studies, with researchers often using a single variable or a limited set of variables that only partly capture the human environment. The overall impact of natural, social, and personal environments on patients with MG remains unclear.

The aim of this study was to systematically investigate the associations between demographic, clinical, and environmental factors and MG severity in a large multicenter cohort from China. We expect that this research will deepen our understanding of MG pathogenesis and inform the management of patients with MG.

## 2 Methods

### 2.1 Study Design and Participants

We conducted a multicenter cross‐sectional study using the national Clinical Cohort Study of MG database. Established in 2017, the project included six representative hospitals across mainland China. Demographic, socioeconomic, residential, lifestyle, and clinical data were collected at registration and during follow‐up to characterize disease features and prognosis in a large cohort. In this analysis, we enrolled 3286 patients with MG who visited participating hospitals between February 2017 and December 2021. MG was diagnosed based on typical myasthenic symptoms and supported by positive autoantibody tests, electrophysiological findings, and/or a neostigmine test [3]. We included patients with an age at onset ≥ 18 years. Patients were excluded if key medical record data were missing (e.g., age at onset, sex, or disease severity scores). The study was approved by the Ethics Committee of Xuanwu Hospital, Capital Medical University ([2017]084). Written informed consent was obtained from all participants. This study followed the Strengthening the Reporting of Observational Studies in Epidemiology (STROBE) reporting guidelines.

### 2.2 Data Collection and Definitions

#### 2.2.1 Demographic and Clinical Characteristics

We obtained the following baseline data from the multicenter database: sex (female, male), age at onset (< 50 years, early‐onset; 50–65 years, late‐onset; ≥ 65 years, very‐late‐onset), symptoms at onset (ocular, generalized), disease duration, comorbidities (yes, no), autoimmune comorbidities (yes, no), thymic features (thymoma, non‐thymoma), history of thymectomy (yes, no), antibody status, and treatment (no therapy, pyridostigmine only, or immunosuppressants). Symptoms at onset were defined as the distribution of muscle weakness within the first month after disease onset. Comorbidities included hypertension, diabetes, and coronary atherosclerotic heart disease. Autoimmune comorbidities were defined as the coexistence of other AIDs, such as autoimmune thyroid disorders, rheumatoid arthritis, and systemic lupus erythematosus. Body mass index (BMI), calculated from height and weight at entry, was also included in the analyses. According to the criteria recommended by the Working Group on Obesity in China (WGOC), BMI was categorized as underweight (< 18.5 kg/m^2^), normal (18.5–23.9 kg/m^2^), and overweight or obesity (≥ 24 kg/m^2^) [10].

#### 2.2.2 Environmental Characteristics

Consistent with prior studies, natural environmental exposures included latitude, altitude, temperature, sunlight duration, and particulate matter with an aerodynamic diameter of ≤ 2.5 μm (PM2.5) [8, 11, 12, 13]. These factors were assigned based on the patients' city of long‐term residence and the calendar year of enrollment. Latitude data were obtained from the National Catalogue Service for Geographic Information website [14]. Given that the natural environment differs substantially between regions at latitudes > 40° N and 30° N–40° N in China, latitude was divided into three regions: low (< 30° N), medium (30° N–40° N), and high (> 40° N) [15]. Altitude data were extracted from the Chinese Environment Database of the Chinese Research Data Services Platform [16]. China's terrain descends from west to east in a three‐step staircase, encompassing Grade I (average altitude > 4000 m), Grade II (1000 m‐2000 m), and Grade III (< 500 m) [17]. Because no patients lived in Grade I regions, we used a modified classification: low (< 500 m), medium (500 m–1000 m), and high (> 1000 m). Annual mean temperature and cumulative sunlight duration were obtained from the Meteorological Yearbook compiled by the China Meteorological Data Service Center [18]. Annual mean PM2.5 concentrations were obtained from the National Tibetan Plateau/Third Pole Environment Data Center [19]. Temperature, sunlight duration, and PM2.5 were categorized into tertiles (low, medium, and high exposure categories) based on their distributions in the cohort. The corresponding cutoff values were as follows: temperature, < 13.9°C, 13.9°C–16.9°C, and > 16.9°C; sunlight duration, < 1619.3 h, 1619.3–2372.3 h, and > 2372.3 h; and PM2.5, < 31.9 μg/m^3^, 31.9–36.8 μg/m^3^, and > 36.8 μg/m^3^.

Socioeconomic status was measured using education, income, and employment status. Based on years of schooling, education level was categorized as less than university (≤ 12 years) and university or above (> 12 years). Income was analyzed at both the individual level (monthly household income) and the area level (per capita disposable income, PDI). Self‐reported monthly household income was classified as low (< ¥3000), medium (¥3000–6000), and high (> ¥6000). Annual PDI data for urban and rural areas across 31 provinces were obtained from the National Bureau of Statistics of China [20] and the values were divided into tertiles. We matched each individual's PDI level to their residence and year of enrollment. Employment status was classified as unemployed (including retirees and students) or employed.

Data on lifestyle factors were also collected at registration. Patients' responses to questions about smoking and alcohol consumption history were recorded as yes or no. Self‐reported physical activity was categorized into three levels: low, medium, and high intensity.

#### 2.2.3 Outcome Measures

Disease severity was assessed at baseline using the MG Activities of Daily Living (MG‐ADL) scale and the Quantitative Myasthenia Gravis (QMG) scale. Based on the classification criteria proposed by Petersson et al. [7], MG‐ADL scores were categorized into two groups: 0–5‐points (mild MG) or ≥ 6 points with at least 1 point from non‐ocular items (moderate to severe MG). Accordingly, patients with a total score of 6 points derived solely from ocular items (*n* = 123) were assigned to the 0–5‐points group, as isolated ocular involvement generally reflects a lower overall disease burden [21, 22]. In line with definitions previously used in clinical trials, QMG scores of 0–10‐points and ≥ 11 points were used to classify patients into mild and moderate to severe MG groups, respectively [23].

### 2.3 Statistical Analysis

Continuous variables were described as median and interquartile range (IQR). Categorical variables were presented as frequency and percentage. Comparisons between groups were analyzed using the Mann–Whitney *U* test for continuous variables and the chi‐square test for categorical variables. Antibody data with more than 20% missing values were excluded from the analysis dataset. Among the variables retained for analysis, eight variables had missing data, with missingness ranging from 3.8% to 12.8% (Table S1). Patterns of missing values and differences in baseline characteristics between complete and incomplete cases were examined (Figure S1 and Table S2). Accordingly, missing data were handled under a missing at random (MAR) assumption using multiple imputation by chained equations. Twenty imputed datasets were created with 10 iterations per imputation. Binary variables were imputed using logistic regression, and ordinal variables were imputed using ordinal logistic regression. No continuous variables had missing data. All variables in Table 1, including MG‐ADL and QMG scores, were included as predictors in the imputation models. To increase statistical power and reduce bias, data were analyzed in each imputed dataset, and the final results were pooled using Rubin's rules [24].

**TABLE 1:** Demographic, clinical, and environmental characteristics of the population according to disease severity.

	Total	MG‐ADL 0–5‐points	MG‐ADL ≥ 6 points		QMG 0–10‐points	QMG ≥ 11 points	
	(*N* = 2468)	(*n* = 1946)	(*n* = 522)	*p*	(*n* = 1992)	(*n* = 476)	*p*
Demographic characteristics							
Female	1330 (53.9)	1017 (52.3)	313 (60.0)	0.002	1029 (51.7)	301 (63.2)	< 0.001
Age at onset, years	49.0 (35.0–60.0)	48.0 (34.0–59.0)	51.5 (39.0–63.3)	< 0.001	48.0 (35.0–59.0)	50.0 (37.0–62.0)	0.006
Age at onset				< 0.001			0.001
Early‐onset (< 50 years)	1298 (52.6)	1058 (54.4)	240 (46.0)		1065 (53.5)	233 (48.9)	
Late‐onset (50–65 years)	764 (31.0)	602 (30.9)	162 (31.0)		626 (31.4)	138 (29.0)	
Very‐late‐onset (≥ 65 years)	406 (16.5)	286 (14.7)	120 (23.0)		301 (15.1)	105 (22.1)	
BMI				< 0.001			0.002
Normal	1055 (45.0)	806 (43.8)	249 (49.6)		831 (44.0)	224 (49.1)	
Underweight	133 (5.7)	84 (4.6)	49 (9.8)		96 (5.1)	37 (8.1)	
Overweight or obesity	1156 (49.3)	952 (51.7)	204 (40.6)		961 (50.9)	195 (42.8)	
Clinical characteristics							
Symptoms at onset				< 0.001			< 0.001
Ocular	1629 (66.0)	1377 (70.8)	252 (48.3)		1395 (70.0)	234 (49.2)	
Generalized	839 (34.0)	569 (29.2)	270 (51.7)		597 (30.0)	242 (50.8)	
Disease duration, months	12.2 (3.3–41.7)	12.8 (3.5–43.6)	9.6 (3.0–35.7)	0.028	12.5 (3.3–43.0)	10.3 (3.2–36.5)	0.423
Comorbidities	649 (26.3)	501 (25.7)	148 (28.4)	0.230	523 (26.3)	126 (26.5)	0.924
Autoimmune comorbidities	172 (7.0)	143 (7.3)	29 (5.6)	0.153	144 (7.2)	28 (5.9)	0.300
Thymoma	563 (24.4)	418 (22.9)	145 (30.0)	0.001	432 (22.9)	131 (31.0)	< 0.001
Thymectomy	512 (20.7)	407 (20.9)	105 (20.1)	0.689	421 (21.1)	91 (19.1)	0.330
Treatment				0.130			0.587
No therapy	488 (19.8)	376 (19.3)	112 (21.5)		390 (19.6)	98 (20.6)	
Pyridostigmine only	673 (27.3)	519 (26.7)	154 (29.5)		537 (27.0)	136 (28.6)	
Immunosuppressant	1307 (53.0)	1051 (54.0)	256 (49.0)		1065 (53.5)	242 (50.8)	
Environmental characteristics							
Latitude				0.001			0.011
Low	619 (25.1)	491 (25.2)	128 (24.5)		517 (26.0)	102 (21.4)	
Medium	1591 (64.5)	1275 (65.5)	316 (60.5)		1282 (64.4)	309 (64.9)	
High	258 (10.5)	180 (9.2)	78 (14.9)		193 (9.7)	65 (13.7)	
Altitude				0.178			0.001
Low	1843 (74.7)	1445 (74.3)	398 (76.2)		1499 (75.3)	344 (72.3)	
Medium	441 (17.9)	361 (18.6)	80 (15.3)		363 (18.2)	78 (16.4)	
High	184 (7.5)	140 (7.2)	44 (8.4)		130 (6.5)	54 (11.3)	
Temperature				0.006			0.215
Low	825 (33.4)	620 (31.9)	205 (39.3)		651 (32.7)	174 (36.6)	
Medium	873 (35.4)	706 (36.3)	167 (32.0)		707 (35.5)	166 (34.9)	
High	770 (31.2)	620 (31.9)	150 (28.7)		634 (31.8)	136 (28.6)	
Sunlight duration				0.018			< 0.001
Low	826 (33.5)	660 (33.9)	166 (31.8)		652 (32.7)	174 (36.6)	
Medium	831 (33.7)	673 (34.6)	158 (30.3)		710 (35.6)	121 (25.4)	
High	811 (32.9)	613 (31.5)	198 (37.9)		630 (31.6)	181 (38.0)	
PM2.5				0.341			0.008
Low	824 (33.4)	636 (32.7)	188 (36.0)		637 (32.0)	187 (39.3)	
Medium	824 (33.4)	654 (33.6)	170 (32.6)		674 (33.8)	150 (31.5)	
High	820 (33.2)	656 (33.7)	164 (31.4)		681 (34.2)	139 (29.2)	
Education				< 0.001			0.001
Less than university	1927 (81.4)	1489 (79.9)	438 (86.7)		1524 (80.1)	403 (86.7)	
University education or above	441 (18.6)	374 (20.1)	67 (13.3)		379 (19.9)	62 (13.3)	
Monthly household income				0.002			< 0.001
Low	522 (22.4)	385 (21.0)	137 (27.8)		390 (20.7)	132 (29.9)	
Medium	741 (31.8)	583 (31.7)	158 (32.1)		593 (31.4)	148 (33.5)	
High	1066 (45.8)	869 (47.3)	197 (40.0)		904 (47.9)	162 (36.7)	
Employment				0.014			0.288
Unemployed	953 (40.2)	725 (38.9)	228 (44.9)		757 (39.6)	196 (42.3)	
Employed	1420 (59.8)	1140 (61.1)	280 (55.1)		1153 (60.4)	267 (57.7)	
Smoking	600 (27.6)	489 (28.5)	111 (24.4)	0.086	489 (27.9)	111 (26.7)	0.624
Alcohol consumption	482 (22.4)	394 (23.1)	88 (19.5)	0.099	396 (22.8)	86 (20.8)	0.396
Physical activity				0.444			0.392
Low	1771 (74.7)	1396 (74.7)	375 (74.7)		1422 (74.6)	349 (75.1)	
Medium	483 (20.4)	386 (20.6)	97 (19.3)		395 (20.7)	88 (18.9)	
High	118 (5.0)	88 (4.7)	30 (6.0)		90 (4.7)	28 (6.0)	

*Note:* Data are presented as number (percentage) or median (interquartile range). The MG‐ADL 0–5‐points group included patients with a score below 6 points or only ocular symptoms. The MG‐ADL ≥ 6 points group included patients with a score of 6 points or higher, with at least 1 point from non‐ocular items.

Abbreviations: BMI, body mass index; MG‐ADL, myasthenia gravis activities of daily living; QMG, quantitative myasthenia gravis score.

Associations between baseline characteristics and disease severity were evaluated using odds ratios (ORs) and 95% confidence intervals (CIs) from univariate and multivariable logistic regression analyses. All variables examined in the univariate analysis were included in the multivariable logistic regression model. The Box‐Tidwell method was used to assess the linearity between continuous independent variables and the logit of the dependent variable. Variance inflation factors (VIF) were computed to confirm the absence of multicollinearity among the independent variables (VIF < 5). Two sensitivity analyses were conducted. First, complete‐case analyses were conducted to assess the robustness of the results from multiple imputation under the MAR assumption. Second, to reduce potential reporting bias, the logistic regression analysis was repeated with monthly household income replaced by PDI. A two‐tailed *p* < 0.05 was considered statistically significant. All analyses were performed using *IBM SPSS Statistics* for Windows, version 26.0 (IBM Corp., Armonk, N.Y., USA).

## 3 Results

**FIGURE 1:**
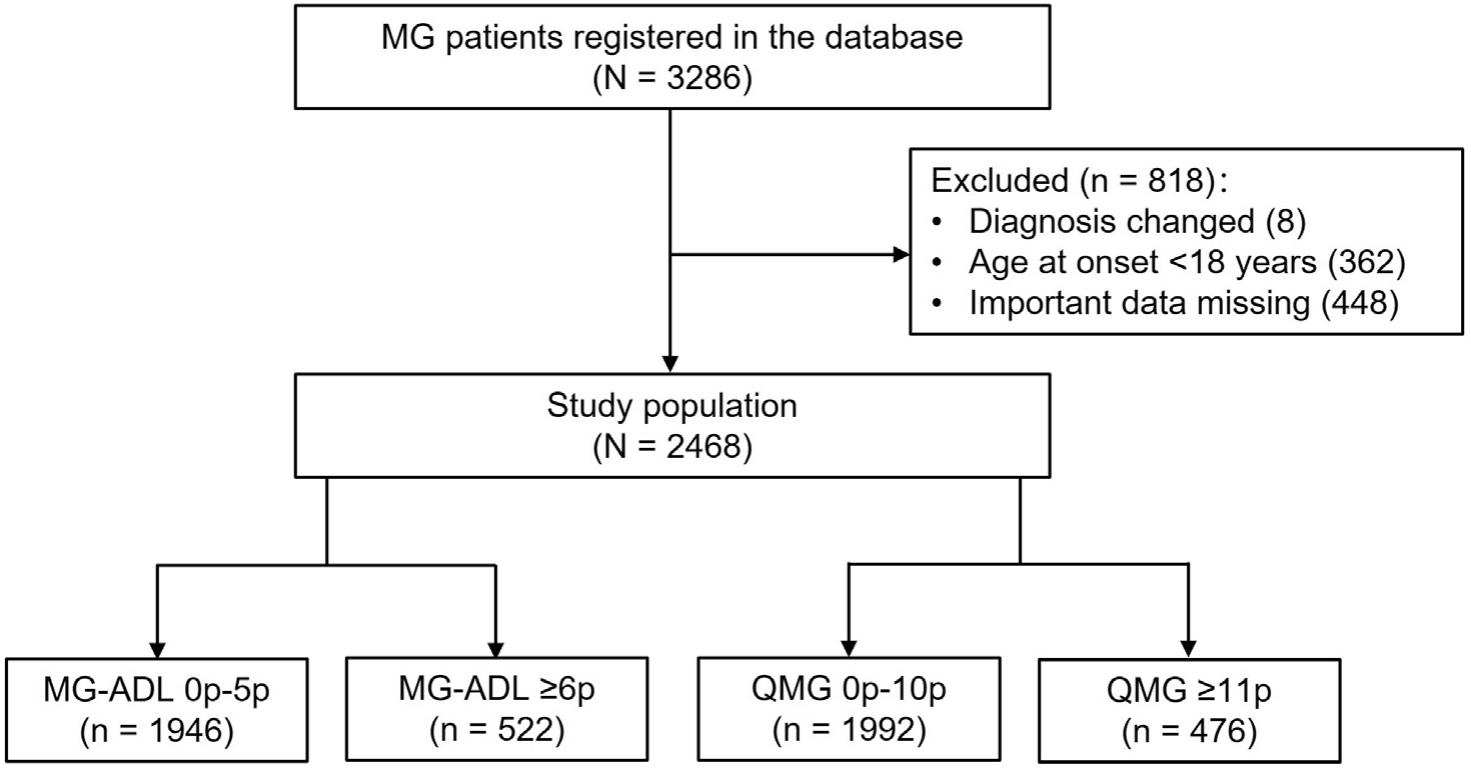
Study flowchart. Patients with an MG‐ADL score of 6 points limited to ocular items were classified into the 0–5‐points group according to the classification proposed by Petersson et al. QMG, quantitative myasthenia gravis score; MG‐ADL, Myasthenia gravis activities of daily living.

**FIGURE 2:**
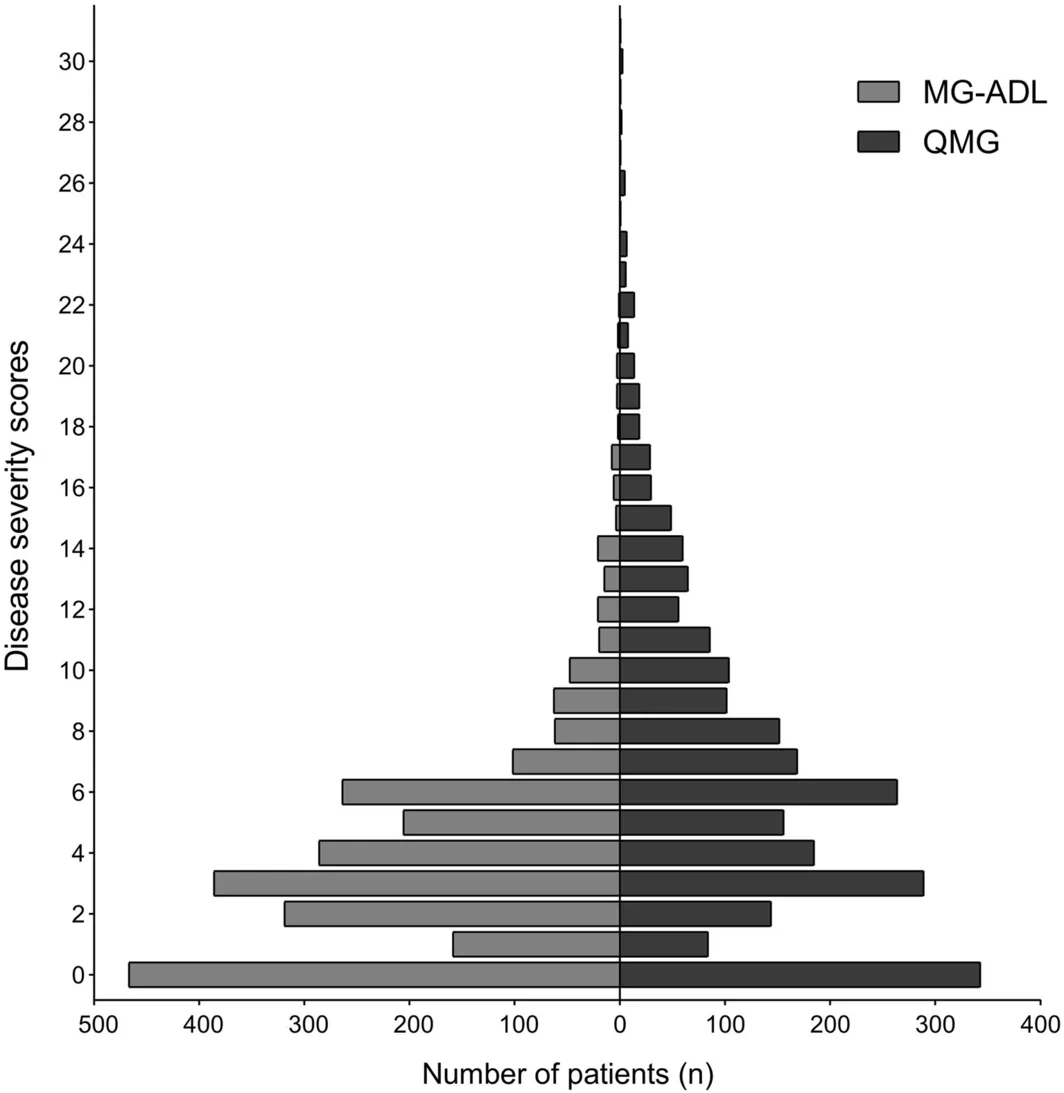
Distribution of disease severity scores in the population. QMG, quantitative myasthenia gravis score; MG‐ADL, Myasthenia gravis activities of daily living.

A total of 2468 patients were included in the final study (Figure 1). The median age at onset was 49.0 years (IQR 35.0–60.0), and 1330 patients (53.9%) were female. The median disease duration at enrollment was 12.2 months (IQR 3.3–41.7). Figure 2 illustrates the distribution of disease severity. The median MG‐ADL score was 3.0 (IQR 1.0–6.0), and the median QMG score was 6.0 (IQR 3.0–9.0). Among the 2468 patients, 522 (21.2%) reported an MG‐ADL score of ≥ 6 points, and 476 (19.3%) had a QMG score of ≥ 11 points, indicating more severe disease (Figure 1).

The baseline characteristics of patients are summarized in Table 1. MG‐ADL scores of ≥ 6 points were more frequently reported by female patients (*p* = 0.002) and those with an older age at onset (*p* < 0.001). Overweight or obese patients were more common in the MG‐ADL 0–5‐points group (*p* < 0.001). Patients with MG‐ADL ≥ 6 points had a higher prevalence of generalized‐onset symptoms (*p* < 0.001), shorter disease duration (*p* = 0.028), and a higher proportion of thymoma (*p* = 0.001) compared with those with MG‐ADL 0–5‐points. Patients with higher MG‐ADL scores were more likely to live at higher latitudes (*p* = 0.001) and to be exposed to lower annual temperatures (*p* = 0.006) and longer sunlight duration (*p* = 0.018). Patients in the MG‐ADL 0–5‐points group had higher education levels (*p* < 0.001), higher monthly household income (*p* = 0.002), and higher employment rates (*p* = 0.014). QMG analyses showed similar patterns, except that disease duration, temperature, and employment status did not differ significantly between groups. Additionally, patients with QMG ≥ 11 points were more likely to live at high altitudes (*p* = 0.001) compared with those with QMG 0–10‐points and were more frequently exposed to lower PM2.5 concentrations (*p* = 0.008).

**FIGURE 3:**
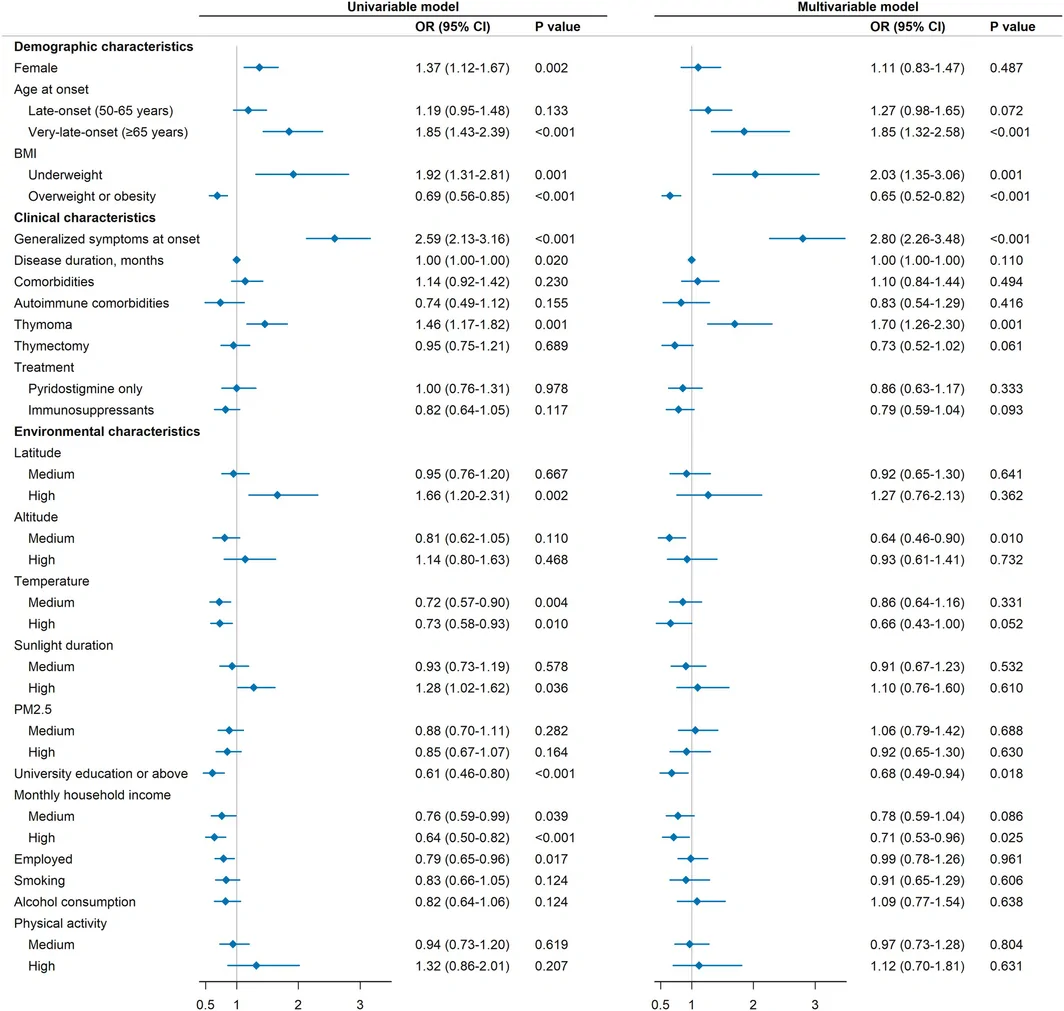
Factors associated with MG‐ADL score ≥ 6 points in patients with myasthenia gravis. MG‐ADL ≥ 6 points required at least 1 point from non‐ocular symptoms. ORs and 95% CIs were based on imputed dataset. Reference groups for the categorical variables are as follows in the order presented in the figure: Male, early‐onset (< 50 years), normal BMI, ocular symptoms at onset, no comorbidities, no autoimmune comorbidities, no thymoma, no thymectomy, no therapy, low latitude, low altitude, low temperature, low sunlight duration, low PM2.5, less than university, low monthly household income, unemployed, no smoking, no alcohol consumption, and low physical activity. BMI, body mass index; CI, confidence interval; OR, odds ratio; PM2.5, particulate matter with an aerodynamic diameter of ≤ 2.5 μm.

**FIGURE 4:**
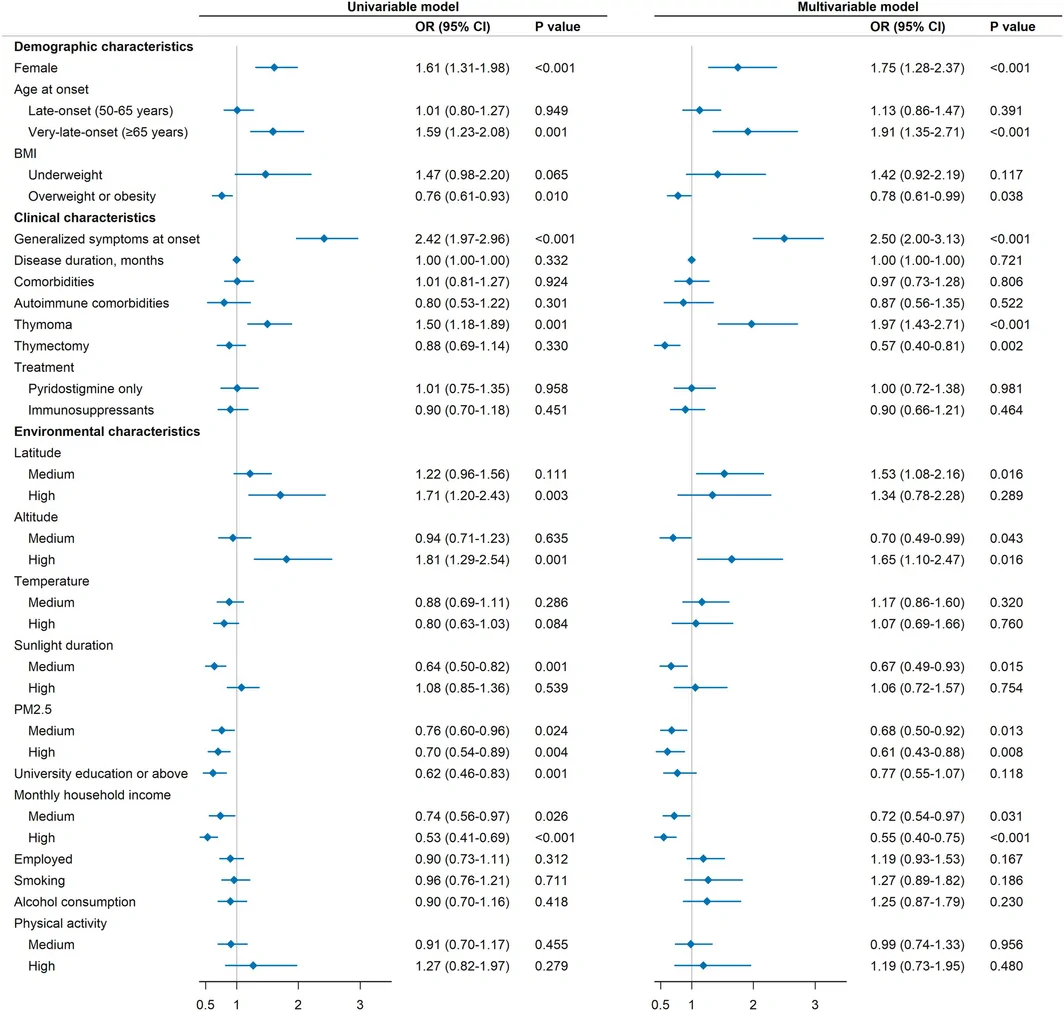
Factors associated with QMG score ≥ 11 points in patients with myasthenia gravis. ORs and 95% CIs were based on imputed dataset. Reference groups for the categorical variables are as follows in the order presented in the figure: Male, early‐onset (< 50 years), normal BMI, ocular symptoms at onset, no comorbidities, no autoimmune comorbidities, no thymoma, no thymectomy, no therapy, low latitude, low altitude, low temperature, low sunlight duration, low PM2.5, less than university, low monthly household income, unemployed, no smoking, no alcohol consumption, and low physical activity. BMI, body mass index; CI, confidence interval; OR, odds ratio; PM2.5, particulate matter with an aerodynamic diameter of ≤ 2.5 μm.

Figure 3 and Figure 4 illustrate the associations between baseline characteristics and disease severity based on multivariable logistic regression models. Age at onset ≥ 65 years (OR 1.85, 95% CI 1.32–2.58), underweight (OR 2.03, 95% CI 1.35–3.06), generalized‐onset symptoms (OR 2.80, 95% CI 2.26–3.48), and thymoma (OR 1.70, 95% CI 1.26–2.30) were significantly associated with MG‐ADL ≥ 6 points, whereas overweight or obesity (OR 0.65, 95% CI 0.52–0.82) was inversely correlated. Medium altitudes (OR 0.64, 95% CI 0.46–0.90), high education levels (OR 0.68, 95% CI 0.49–0.94), and high monthly household income (OR 0.71, 95% CI 0.53–0.96) were associated with lower MG‐ADL scores. Results for the QMG scale were partially consistent with the findings above and provided additional insights (Figure 4). Female sex (OR 1.75, 95% CI 1.28–2.37), medium latitudes (OR 1.53, 95% CI 1.08–2.16), and high altitudes (OR 1.65, 95% CI 1.10–2.47) were associated with QMG ≥ 11 points. Conversely, thymectomy (OR 0.57, 95% CI 0.40–0.81), medium sunlight duration (OR 0.67, 95% CI 0.49–0.93), and higher PM2.5 concentrations were associated with lower QMG scores. There were no significant associations between disease duration, comorbidities, autoimmune comorbidities, treatment, temperature, employment, smoking, alcohol consumption, or physical activity and MG‐ADL or QMG severity.

In the complete‐case sensitivity analysis, the results were consistent with the primary analysis (Tables S3 and S4). Similar findings were observed when self‐reported income was replaced with provincial‐level PDI (Tables S5 and S6). Despite the observed inverse association between temperature and MG‐ADL scores, similar effect sizes in the primary and sensitivity analyses supported the robustness of the primary findings.

## 4 Discussion

Our multicenter cross‐sectional study investigated the associations between demographic, clinical, and environmental factors and MG severity, as measured by the MG‐ADL and QMG scales. The study found that sex, age at onset, BMI, onset symptoms, thymoma, and thymectomy were correlated with disease scores. Environmental exposures, including latitude, altitude, sunlight duration, PM2.5, education, and monthly household income, were also associated with MG severity.

Women are generally more susceptible to AIDs. Our study found that female sex was significantly associated with higher QMG scores, consistent with previous reports [4, 7]. This association may be attributed to the role of estrogen in regulating autoimmune responses [25]. We also observed that age at onset ≥ 65 years was correlated with moderate to severe disease. A prior study from China reported similar findings and noted a poorer prognosis in these patients than in younger individuals [26]. However, other research has shown contrasting results. A large population‐based study from Spain indicated that patients with very‐late‐onset MG had favorable outcomes when treated appropriately, even though they experienced more life‐threatening events at disease onset [27]. Cutter et al. also found that the very‐late‐onset group reported better scores on disease‐specific quality of life and lower disease severity [28]. Differences in immunotherapy regimens and follow‐up duration across studies may account for these discrepancies [26].

Patients with MG are more likely to be overweight or obese than the general population [29], possibly due to reduced physical activity or steroid‐related side effects. A high BMI is typically associated with worse disease status, reduced quality of life, and adverse postoperative outcomes in MG [7, 22, 30]. However, contrary to previous studies, overweight or obese patients in our cohort had a lower risk of moderate to severe symptoms at baseline, potentially indicating an “obesity paradox” [31]. Several mechanisms could explain this finding. First, adiponectin, an adipocytokine produced by adipocytes, has been linked to proinflammatory effects in various AIDs, but its levels decrease with weight gain [32]. This supports observations that obese patients with early rheumatoid arthritis had less joint damage than those with normal weight [33, 34], consistent with our findings. Second, obesity may contribute to earlier fatigue and respiratory symptoms, which may facilitate earlier clinical recognition and treatment, thereby improving outcomes. Third, the limitations of BMI in assessing body composition and fat distribution may obscure the actual effects of being overweight or obese [31]. Further longitudinal studies tracking weight changes and disease progression are needed to elucidate the impact of BMI on MG.

While much is known about the influence of demographic and clinical characteristics, fewer studies have explored the association between environmental factors and MG. Sunlight duration, a crude proxy for ultraviolet radiation (UVR) exposure, was associated with disease severity in our study. Patients in the medium sunlight duration group had lower QMG scores, which may be explained by enhanced UVR exposure and vitamin D synthesis. Previous reports have shown that vitamin D deficiency is associated with higher MG‐ADL scores, while vitamin D supplementation may help attenuate muscle weakness [35, 36]. Consistent with this hypothesis, the lower disease severity observed among patients living at medium altitudes may be related to greater UVR exposure, whereas the higher QMG scores observed in patients living at medium latitudes may reflect lower UVR exposure and reduced immunomodulatory benefits [11]. However, the association between high altitude and greater QMG scores suggests that the effects of UVR may vary across exposure levels. Excessive exposure at higher altitudes may activate immune pathways that are unfavorable for disease control. Studies in multiple sclerosis have shown that UVR can upregulate Type I interferon in a vitamin D‐independent manner [37], and this cytokine has been implicated in MG pathogenesis and antibody production [38]. In addition, chronic hypobaric hypoxia at high altitudes may contribute to systemic inflammatory activation and potentially increase disease severity [39]. Nevertheless, other unmeasured factors, including regional genetic background, sun‐protection behaviors, and outdoor activity patterns, could also play a role and warrant further investigation [40].

The inverse association between PM2.5 and QMG scores in our study is counterintuitive and contrasts with findings in other AIDs. One plausible explanation is residual confounding, particularly by urbanization level and SES (e.g., economic activity, healthcare access, population density, occupational exposures, and housing conditions) [41]. These factors are difficult to fully capture and may bias estimates of the air pollution effect [42]. Our hospital‐based recruitment may also have introduced selection bias. Due to limited healthcare accessibility, patients from relatively cleaner areas (often less urbanized) may be referred to a tertiary center only after symptoms become severe. In contrast, patients living in more urbanized and polluted areas may receive earlier diagnosis and treatment, leading to lower disease severity at enrollment. Additionally, regional PM2.5 assignments may not accurately reflect individual long‐term exposure, as residential mobility and time‐activity patterns vary among individuals [43]. Taken together, the observed inverse association should be considered exploratory and hypothesis‐generating. Future longitudinal studies with better exposure assessment and confounder control are warranted to clarify the relationship between PM2.5 exposure and MG prognosis.

Socioeconomic status, often assessed by factors such as income level, educational attainment, and occupation, has been shown to affect the onset and prognosis of many AIDs [44]. In our study, the association between university education and MG‐ADL scores was consistent with that reported by Petersson et al. [7]. The protective effect of higher educational attainment may be driven by more active healthcare‐seeking behavior and healthier lifestyle choices [7, 41]. In multivariable analyses, higher household income was correlated with improved disease status, and this association remained when PDI was used as a less biased indicator. These results also align with our previous cohort study, in which ocular MG patients living in areas with higher PDI had a lower risk of generalization [6]. Compared with high‐income groups, low‐income patients may experience delayed diagnosis and treatment, irregular follow‐up, and poor medication adherence, which could contribute to worse outcomes. Our findings highlight the impact of SES on MG and have implications for health policy [44]. Efforts are needed to reduce disparities in access to quality care and higher education across social groups.

The major strength of our study is the large sample size derived from a nationwide multicenter cohort, which enhances the generalizability of the findings. In contrast to prior studies that used single or limited variables, we constructed multivariable models that incorporated natural, social, and personal domains. This approach allows a comprehensive evaluation of environmental factors associated with MG. Additionally, we used both objective and subjective measures to assess muscle weakness, which helped address the limited sensitivity of the MG‐ADL scale in detecting mild symptoms. The use of both scales enables a more accurate measurement of disease severity and provides additional clinical information.

This study had several limitations. First, the cross‐sectional design precluded causal inference between factors and disease severity, particularly for variables that may be influenced by MG (e.g., BMI, income, and employment status). Therefore, these findings should be interpreted cautiously. Second, all patients were recruited from top‐ranked hospitals, which may have overestimated the proportion of severe cases. However, the median MG‐ADL score in our cohort was lower than that reported in previous studies [7, 28], suggesting that selection bias may be limited. Third, because detailed residential history (e.g., the specific locations and duration of past residences) was not available, environmental exposures were assigned according to the city of long‐term residence reported at enrollment. This may introduce some misclassification of long‐term exposure, particularly among patients who recently moved. Fourth, most data were self‐reported, which may lead to recall and measurement bias, thereby reducing the accuracy of the information. Fifth, missing data were present, which may affect the association between variables and disease severity. However, the multiple imputation approach used in our study may have helped to reduce potential bias. Complete‐case sensitivity analyses also supported the robustness of our findings. Finally, pathogenic antibody status was excluded from the analyses due to a high proportion of missing values, which may have resulted in residual confounding. Data on other environmental determinants, such as diet, marital status, and social support, were not available and should be considered in future studies [8, 45].

## 5 Conclusion

This multicenter cross‐sectional study systematically evaluated the associations of demographic, clinical, and environmental factors with MG severity. Female patients and those with very‐late‐onset MG, underweight, generalized‐onset symptoms, or thymoma had higher disease scores, whereas overweight or obesity and thymectomy played a protective role. Environmental exposures, including latitude, altitude, sunlight duration, PM2.5, education, and monthly household income, were also associated with disease severity. Our findings support further investigation into the role of environmental factors in MG pathogenesis, which is important for disease management and public health policy.

## Author Contributions

Haoran Liu, Jingsi Wang, and Wenjia Zhu designed the study. Haoran Liu conducted the statistical analysis, interpreted the results, and drafted the manuscript with the help of Jingsi Wang. Wenjia Zhu, Ting Chang, Hongxi Chen, Chao Sun, Rongjing Guo, Zhe Ruan, Fangfang Bi, Jing Li, Jianwen Wang, Shu Zhang, Qi Wen, Yaye Wang, Yun Li, Nairong Xie, Yuting Jiang, Yan Lu, Li Di, Min Xu, Min Wang, Hai Chen, Xinmei Wen, Jianying Duo, Yue Huang, Huiyu Feng, Hongyu Zhou, and Yuwei Da carried out data collection, investigation, and project administration. Haoran Liu, Huiyu Feng, Hongyu Zhou, and Yuwei Da reviewed and edited the article. All authors read and approved the final manuscript.

## Funding

This work was supported by the National Natural Science Foundation of China (82571595, 62171299) and the Clinical Cohort Study of Myasthenia Gravis, National Key R&D Program of China, Precision Medicine Project (2017YFC0907700).

## Ethics Statement

The study was approved by the Ethics Committee of Xuanwu Hospital, Capital Medical University ([2017]084), with all participants providing written informed consent before enrollment.

## Conflicts of Interest

The authors declare no conflicts of interest.

## Supporting information


**Table S1:** Information on missing data in the study.
**Table S2:** Baseline characteristics of complete and incomplete cases defined by missingness.
**Table S3:** Factors influencing risk of myasthenia gravis activities of daily living score (MG‐ADL) ≥ 6p.
**Table S4:** Factors influencing risk of quantitative myasthenia gravis score (QMG) ≥ 11p.
**Table S5:** Factors influencing risk of myasthenia gravis activities of daily living score (MG‐ADL) ≥ 6p.
**Table S6:** Factors influencing risk of quantitative myasthenia gravis score (QMG) ≥ 11p.
Figure. S1 Missingness map.

## Data Availability

The data that support the findings of this study are available from the corresponding author upon reasonable request.
