# Supplementary material for: Association of Demographic, Clinical, and Environmental Factors With Disease Severity in Adult Myasthenia Gravis: A Multicenter Cross‐Sectional Study
Source: CNS Neurosci Ther. 2026 Aug 25;32(8):e71104. doi: 10.1002/cns.71104 (PMC13504371; doi:10.1002/cns.71104)
Supplement: Supplementary file 1 [file CNS-32-e71104-s001.docx]

**Supplementary material**

| **Table S1** Information on missing data in the study | | |
| --- | --- | --- |
|  | **Total** | **Missing (%)** |
| Alcohol consumption | 2153 | 315 (12.8%) |
| Smoking | 2170 | 298 (12.1%) |
| Thymoma | 2305 | 163 (6.6%) |
| Monthly household income | 2329 | 139 (5.6%) |
| Body-mass index | 2344 | 124 (5.0%) |
| Education | 2368 | 100 (4.4%) |
| Physical activity | 2372 | 96 (3.9%) |
| Employment | 2373 | 95 (3.8%) |
| Other variables had no missing data. | | |

| **Table S2** Baseline characteristics of complete and incomplete cases defined by missingness | | |
| --- | --- | --- |
| **Variable** | **Incomplete cases (n=725)** | **Complete cases (n=1743)** |
| Female | 432 (59.6) | 898 (51.5) |
| Age at onset |  |  |
| Early-onset (<50 years) | 441 (60.8) | 857 (49.2) |
| Late-onset (50-65 years) | 200 (27.6) | 564 (32.4) |
| Very-late-onset (≥65 years) | 84 (11.6) | 322 (18.5) |
| Symptoms at onset |  |  |
| Ocular | 380 (52.4) | 1249 (71.7) |
| Generalized | 345 (47.6) | 494 (28.3) |
| Disease duration, months | 10.2 (3.1, 36.5) | 17.3 (4.2, 52.1) |
| Comorbidities | 132 (18.2) | 517 (29.7) |
| Autoimmune comorbidities | 61 (8.4) | 111 (6.4) |
| Thymectomy | 168 (23.2) | 344 (19.7) |
| Treatment |  |  |
| No therapy | 140 (19.3) | 348 (20.0) |
| Pyridostigmine only | 134 (18.5) | 539 (30.9) |
| Immunosuppressant | 451 (62.2) | 856 (49.1) |
| Latitude |  |  |
| Low | 278 (38.3) | 341 (19.6) |
| Medium | 429 (59.2) | 1162 (66.7) |
| High | 18 (2.5) | 240 (13.8) |
| Altitude |  |  |
| Low | 487 (67.2) | 1356 (77.8) |
| Medium | 190 (26.2) | 251 (14.4) |
| High | 48 (6.6) | 136 (7.8) |
| Temperature |  |  |
| Low | 143 (19.7) | 682 (39.1) |
| Medium | 260 (35.9) | 613 (35.2) |
| High | 322 (44.4) | 448 (25.7) |
| Sunlight duration |  |  |
| Low | 404 (55.7) | 422 (24.2) |
| Medium | 249 (34.3) | 582 (33.4) |
| High | 72 (9.9) | 739 (42.4) |
| PM2.5 |  |  |
| Low | 285 (39.3) | 539 (30.9) |
| Medium | 278 (38.3) | 546 (31.3) |
| High | 162 (22.3) | 162 (37.8) |
| MG-ADL ≥6p^a^ | 159 (21.9) | 486 (27.9) |
| QMG ≥11p | 135 (18.6) | 341 (19.6) |
| Abbreviations: MG-ADL = myasthenia gravis activities of daily living; QMG = quantitative myasthenia gravis score  ^a^ MG-ADL ≥6p required at least 1p from non-ocular symptoms.  Complete cases were defined as patients with no missing values in any variables. Incomplete cases were defined as patients with at least one missing value. | | |

| **Table S3** Factors influencing risk of myasthenia gravis activities of daily living score (MG-ADL) ≥6p^a^ | | | | |
| --- | --- | --- | --- | --- |
|  | **Univariable model** | | **Multivariable model^b^** | |
|  | **OR (95% CI)** | **P value** | **OR (95% CI)** | **P value** |
| **Demographic characteristics** |  |  |  |  |
| Female | 1.55 (1.23-1.95) | **<0.001** | 1.15 (0.83-1.59) | 0.393 |
| Age at onset |  |  |  |  |
| Early-onset (<50 years) | 1 (Reference) |  | 1 (Reference) |  |
| Late-onset (50-65 years) | 1.10 (0.84-1.43) | 0.490 | 1.24 (0.91-1.69) | 0.174 |
| Very-late-onset (≥65 years) | 1.89 (1.41-2.53) | **<0.001** | 2.03 (1.38-2.98) | **<0.001** |
| BMI |  |  |  |  |
| Normal | 1 (Reference) |  | 1 (Reference) |  |
| Underweight | 2.05 (1.26-3.32) | **0.004** | 2.30 (1.36-3.90) | **0.002** |
| Overweight or obesity | 0.70 (0.55-0.89) | **0.003** | 0.69 (0.53-0.90) | **0.006** |
| **Clinical characteristics** |  |  |  |  |
| Generalized symptoms at onset | 2.60 (2.05-3.30) | **<0.001** | 2.71 (2.09-3.51) | **<0.001** |
| Disease duration, months | 1.00 (1.00-1.00) | 0.350 | 1.00 (1.00-1.00) | 0.901 |
| Comorbidities | 1.08 (0.84-1.38) | 0.552 | 0.95 (0.70-1.29) | 0.730 |
| Autoimmune comorbidities | 0.77 (0.47-1.27) | 0.306 | 0.83 (0.48-1.40) | 0.477 |
| Thymoma | 1.38 (1.07-1.79) | **0.014** | 1.45 (1.03-2.06) | **0.036** |
| Thymectomy | 1.08 (0.81-1.43) | 0.600 | 0.88 (0.60-1.30) | 0.516 |
| Treatment |  |  |  |  |
| No therapy | 1 (Reference) |  | 1 (Reference) |  |
| Pyridostigmine only | 1.01 (0.73-1.38) | 0.972 | 0.93 (0.65-1.33) | 0.686 |
| Immunosuppressant | 0.85 (0.63-1.14) | 0.277 | 0.91 (0.65-1.27) | 0.577 |
| **Environmental characteristics** |  |  |  |  |
| Latitude |  |  |  |  |
| Low | 1 (Reference) |  | 1 (Reference) |  |
| Medium | 1.51 (1.09-2.08) | **0.012** | 1.71 (1.06-2.77) | **0.029** |
| High | 2.23 (1.49-3.34) | **<0.001** | 1.86 (0.98-3.53) | 0.058 |
| Altitude |  |  |  |  |
| Low | 1 (Reference) |  | 1 (Reference) |  |
| Medium | 0.83 (0.59-1.17) | 0.290 | 0.62 (0.41-0.93) | **0.023** |
| High | 1.17 (0.78-1.77) | 0.443 | 0.90 (0.56-1.46) | 0.681 |
| Temperature |  |  |  |  |
| Low | 1 (Reference) |  | 1 (Reference) |  |
| Medium | 0.68 (0.52-0.88) | **0.004** | 0.77 (0.56-1.08) | 0.126 |
| High | 0.63 (0.47-0.84) | **0.002** | 0.76 (0.44-1.32) | 0.324 |
| Sunlight duration |  |  |  |  |
| Low | 1 (Reference) |  | 1 (Reference) |  |
| Medium | 1.08 (0.78-1.48) | 0.652 | 1.04 (0.70-1.56) | 0.833 |
| High | 1.43 (1.06-1.92) | **0.018** | 1.16 (0.73-1.85) | 0.524 |
| PM2.5 |  |  |  |  |
| Low | 1 (Reference) |  | 1 (Reference) |  |
| Medium | 0.89 (0.67-1.19) | 0.441 | 1.00 (0.69-1.46) | 0.988 |
| High | 0.90 (0.69-1.19) | 0.466 | 0.82 (0.53-1.27) | 0.369 |
| University education or above | 0.48 (0.34-0.68) | **<0.001** | 0.58 (0.39-0.85) | **0.005** |
| Monthly household income |  |  |  |  |
| Low | 1 (Reference) |  | 1 (Reference) |  |
| Medium | 0.67 (0.50-0.90) | **0.008** | 0.73 (0.53-1.01) | 0.057 |
| High | 0.55 (0.42-0.73) | **<0.001** | 0.71 (0.51-0.99) | **0.043** |
| Employed | 0.77 (0.61-0.97) | **0.027** | 1.00 (0.76-1.32) | 0.998 |
| Smoking | 0.76 (0.58-0.99) | **0.041** | 0.87 (0.60-1.27) | 0.478 |
| Alcohol consumption | 0.74 (0.56-0.98) | **0.036** | 1.05 (0.71-1.54) | 0.821 |
| Physical activity |  |  |  |  |
| Low | 1 (Reference) |  | 1 (Reference) |  |
| Medium | 0.90 (0.67-1.21) | 0.486 | 0.98 (0.70-1.37) | 0.907 |
| High | 1.21 (0.75-1.96) | 0.438 | 1.11 (0.65-1.90) | 0.710 |
| Abbreviations: OR = odds ratio; CI = confidence interval; BMI = body mass index.  ^a^ MG-ADL ≥6p required at least 1p from non-ocular symptoms.  ^b^ ORs and 95% CIs were based on complete dataset. Bold values are statistically significant (p < 0.05). | | | | |

| **Table S4** Factors influencing risk of quantitative myasthenia gravis score (QMG) ≥11p | | | | |
| --- | --- | --- | --- | --- |
|  | **Univariable model** | | **Multivariable model^a^** | |
|  | **OR (95% CI)** | **P value** | **OR (95% CI)** | **P value** |
| **Demographic characteristics** |  |  |  |  |
| Female | 1.88 (1.47-2.40) | **<0.001** | 1.86 (1.30-2.65) | **0.001** |
| Age at onset |  |  |  |  |
| Early-onset (<50 years) | 1 (Reference) |  | 1 (Reference) |  |
| Late-onset (50-65 years) | 0.90 (0.68-1.19) | 0.448 | 1.03 (0.75-1.42) | 0.854 |
| Very-late-onset (≥65 years) | 1.50 (1.11-2.03) | **0.009** | 1.84 (1.23-2.75) | **0.003** |
| BMI |  |  |  |  |
| Normal | 1 (Reference) |  | 1 (Reference) |  |
| Underweight | 1.31 (0.77-2.22) | 0.319 | 1.27 (0.72-2.26) | 0.410 |
| Overweight or obesity | 0.73 (0.57-0.93) | **0.010** | 0.79 (0.60-1.04) | 0.095 |
| **Clinical characteristics** |  |  |  |  |
| Generalized symptoms at onset | 2.65 (2.08-3.39) | **<0.001** | 2.68 (2.05-3.50) | **<0.001** |
| Disease duration, months | 1.00 (1.00-1.00) | 0.624 | 1.00 (1.00-1.00) | 0.733 |
| Comorbidities | 0.96 (0.74-1.25) | 0.777 | 0.88 (0.64-1.22) | 0.450 |
| Autoimmune comorbidities | 0.90 (0.55-1.48) | 0.671 | 0.95 (0.56-1.62) | 0.850 |
| Thymoma | 1.46 (1.12-1.90) | **0.005** | 1.82 (1.27-2.61) | **0.001** |
| Thymectomy | 0.97 (0.72-1.31) | 0.844 | 0.63 (0.42-0.96) | **0.031** |
| Treatment |  |  |  |  |
| No therapy | 1 (Reference) |  | 1 (Reference) |  |
| Pyridostigmine | 0.97 (0.69-1.35) | 0.842 | 1.06 (0.73-1.56) | 0.751 |
| Immunosuppressant | 0.92 (0.67-1.25) | 0.589 | 1.17 (0.82-1.68) | 0.389 |
| **Environmental characteristics** |  |  |  |  |
| Latitude |  |  |  |  |
| Low | 1 (Reference) |  | 1 (Reference) |  |
| Medium | 1.96 (1.37-2.81) | **<0.001** | 3.04 (1.84-5.03) | **<0.001** |
| High | 2.56 (1.65-3.98) | **<0.001** | 2.16 (1.11-4.23) | **0.024** |
| Altitude |  |  |  |  |
| Low | 1 (Reference) |  | 1 (Reference) |  |
| Medium | 0.87 (0.61-1.24) | 0.432 | 0.65 (0.42-1.01) | 0.053 |
| High | 1.57 (1.05-2.34) | **0.028** | 1.23 (0.76-1.99) | 0.409 |
| Temperature |  |  |  |  |
| Low | 1 (Reference) |  | 1 (Reference) |  |
| Medium | 0.82 (0.63-1.08) | 0.153 | 1.09 (0.77-1.54) | 0.640 |
| High | 0.70 (0.51-0.95) | **0.021** | 1.17 (0.66-2.08) | 0.589 |
| Sunlight duration |  |  |  |  |
| Low | 1 (Reference) |  | 1 (Reference) |  |
| Medium | 0.85 (0.61-1.18) | 0.322 | 0.89 (0.58-1.36) | 0.587 |
| High | 1.27 (0.94-1.71) | 0.120 | 1.29 (0.80-2.10) | 0.303 |
| PM2.5 |  |  |  |  |
| Low | 1 (Reference) |  | 1 (Reference) |  |
| Medium | 0.63 (0.47-0.85) | **0.003** | 0.50 (0.33-0.74) | **0.001** |
| High | 0.76 (0.58-1.01) | 0.054 | 0.51 (0.32-0.80) | **0.004** |
| University education or above | 0.57 (0.41-0.80) | **0.001** | 0.75 (0.51-1.11) | 0.148 |
| Monthly household income |  |  |  |  |
| Low | 1 (Reference) |  | 1 (Reference) |  |
| Medium | 0.69 (0.51-0.94) | **0.018** | 0.73 (0.52-1.02) | 0.061 |
| High | 0.51 (0.38-0.69) | **<0.001** | 0.61 (0.43-0.86) | **0.005** |
| Employed | 0.93 (0.73-1.18) | 0.562 | 1.18 (0.88-1.58) | 0.263 |
| Smoking | 0.81 (0.61-1.06) | 0.121 | 1.05 (0.70-1.58) | 0.803 |
| Alcohol consumption | 0.81 (0.61-1.09) | 0.159 | 1.33 (0.88-1.99) | 0.176 |
| Physical activity |  |  |  |  |
| Low | 1 (Reference) |  | 1 (Reference) |  |
| Medium | 0.97 (0.71-1.30) | 0.817 | 1.12 (0.79-1.59) | 0.523 |
| High | 1.26 (0.77-2.08) | 0.355 | 1.28 (0.73-2.25) | 0.394 |
| Abbreviations: OR = odds ratio; CI = confidence interval; BMI = body mass index.  ^a^ ORs and 95% CIs were based on complete dataset. Bold values are statistically significant (p < 0.05). | | | | |
| **Table S5** Factors influencing risk of myasthenia gravis activities of daily living score (MG-ADL) ≥6p^a^ | | | | |
|  | **Univariable model** | | **Multivariable model^b^** | |
|  | **OR (95% CI)** | **P value** | **OR (95% CI)** | **P value** |
| **Demographic characteristics** |  |  |  |  |
| Female | 1.37 (1.12-1.67) | **0.002** | 1.08 (0.82-1.44) | 0.580 |
| Age at onset |  |  |  |  |
| Early-onset (<50 years) | 1 (Reference) |  | 1 (Reference) |  |
| Late-onset (50-65 years) | 1.19 (0.95-1.48) | 0.133 | 1.27 (0.98-1.65) | 0.069 |
| Very-late-onset (≥65 years) | 1.85 (1.43-2.39) | **<0.001** | 1.87 (1.34-2.62) | **<0.001** |
| BMI |  |  |  |  |
| Normal | 1 (Reference) |  | 1 (Reference) |  |
| Underweight | 1.92 (1.31-2.81) | **0.001** | 2.02 (1.34-3.04) | **0.001** |
| Overweight or obesity | 0.69 (0.56-0.85) | **<0.001** | 0.65 (0.52-0.82) | **<0.001** |
| **Clinical characteristics** |  |  |  |  |
| Generalized symptoms at onset | 2.59 (2.13-3.16) | **<0.001** | 2.80 (2.26-3.48) | **<0.001** |
| Disease duration, months | 1.00 (1.00-1.00) | **0.020** | 1.00 (1.00-1.00) | 0.134 |
| Comorbidities | 1.14 (0.92-1.42) | 0.230 | 1.10 (0.84-1.44) | 0.485 |
| Autoimmune comorbidities | 0.74 (0.49-1.12) | 0.155 | 0.85 (0.55-1.31) | 0.460 |
| Thymoma | 1.46 (1.17-1.82) | **0.001** | 1.69 (1.25-2.28) | **0.001** |
| Thymectomy | 0.95 (0.75-1.21) | 0.689 | 0.74 (0.53-1.03) | 0.070 |
| Treatment |  |  |  |  |
| No therapy | 1 (Reference) |  | 1 (Reference) |  |
| Pyridostigmine only | 1.00 (0.76-1.31) | 0.978 | 0.85 (0.62-1.15) | 0.289 |
| Immunosuppressant | 0.82 (0.64-1.05) | 0.117 | 0.79 (0.59-1.05) | 0.099 |
| **Environmental characteristics** |  |  |  |  |
| Latitude |  |  |  |  |
| Low | 1 (Reference) |  | 1 (Reference) |  |
| Medium | 0.95 (0.76-1.20) | 0.667 | 0.95 (0.67-1.34) | 0.775 |
| High | 1.66 (1.20-2.31) | **0.002** | 1.34 (0.81-2.24) | 0.257 |
| Altitude |  |  |  |  |
| Low | 1 (Reference) |  | 1 (Reference) |  |
| Medium | 0.81 (0.62-1.05) | 0.110 | 0.64 (0.46-0.90) | **0.011** |
| High | 1.14 (0.80-1.63) | 0.468 | 0.90 (0.59-1.37) | 0.626 |
| Temperature |  |  |  |  |
| Low | 1 (Reference) |  | 1 (Reference) |  |
| Medium | 0.72 (0.57-0.90) | **0.004** | 0.88 (0.65-1.19) | 0.401 |
| High | 0.73 (0.58-0.93) | **0.010** | 0.65 (0.42-0.99) | **0.042** |
| Sunlight duration |  |  |  |  |
| Low | 1 (Reference) |  | 1 (Reference) |  |
| Medium | 0.93 (0.73-1.19) | 0.578 | 0.89 (0.66-1.20) | 0.442 |
| High | 1.28 (1.02-1.62) | **0.036** | 1.11 (0.76-1.60) | 0.597 |
| PM2.5 |  |  |  |  |
| Low | 1 (Reference) |  | 1 (Reference) |  |
| Medium | 0.88 (0.70-1.11) | 0.282 | 1.06 (0.79-1.42) | 0.700 |
| High | 0.85 (0.67-1.07) | 0.164 | 0.92 (0.65-1.30) | 0.630 |
| University education or above | 0.61 (0.46-0.80) | **<0.001** | 0.66 (0.49-0.91) | **0.011** |
| PDI |  |  |  |  |
| Low | 1 (Reference) |  | 1 (Reference) |  |
| Medium | 0.79 (0.58-1.08) | 0.141 | 0.79 (0.56-1.10) | 0.164 |
| High | 0.65 (0.50-0.83) | **0.001** | 0.69 (0.51-0.92) | **0.012** |
| Employed | 0.79 (0.65-0.96) | **0.017** | 0.97 (0.76-1.24) | 0.804 |
| Smoking | 0.83 (0.66-1.05) | 0.124 | 0.91 (0.65-1.28) | 0.590 |
| Alcohol consumption | 0.82 (0.64-1.06) | 0.124 | 1.07 (0.75-1.50) | 0.723 |
| Physical activity |  |  |  |  |
| Low | 1 (Reference) |  | 1 (Reference) |  |
| Medium | 0.94 (0.73-1.20) | 0.619 | 0.95 (0.72-1.26) | 0.713 |
| High | 1.32 (0.86-2.01) | 0.207 | 1.11 (0.68-1.78) | 0.683 |
| Abbreviations: OR = odds ratio; CI = confidence interval; BMI = body mass index; PDI = per capita disposable income.  ^a^ MG-ADL ≥6p required at least 1p from non-ocular symptoms.  ^b^ ORs and 95% CIs were based on imputed dataset. Bold values are statistically significant (p < 0.05). | | | | |

| **Table S6** Factors influencing risk of quantitative myasthenia gravis score (QMG) ≥11p | | | | |
| --- | --- | --- | --- | --- |
|  | **Univariable model** | | **Multivariable model^a^** | |
|  | **OR (95% CI)** | **P value** | **OR (95% CI)** | **P value** |
| **Demographic characteristics** |  |  |  |  |
| Female | 1.61 (1.31-1.98) | **<0.001** | 1.70 (1.25-2.31) | **0.001** |
| Age at onset |  |  |  |  |
| Early-onset (<50 years) | 1 (Reference) |  | 1 (Reference) |  |
| Late-onset (50-65 years) | 1.01 (0.80-1.27) | 0.949 | 1.12 (0.86-1.47) | 0.410 |
| Very-late-onset (≥65 years) | 1.59 (1.23-2.08) | **0.001** | 1.97 (1.39-2.79) | **<0.001** |
| BMI |  |  |  |  |
| Normal | 1 (Reference) |  | 1 (Reference) |  |
| Underweight | 1.47 (0.98-2.20) | 0.065 | 1.41 (0.92-2.19) | 0.118 |
| Overweight or obesity | 0.76 (0.61-0.93) | **0.010** | 0.79 (0.62-1.00) | **0.048** |
| **Clinical characteristics** |  |  |  |  |
| Generalized symptoms at onset | 2.42 (1.97-2.96) | **<0.001** | 2.50 (2.00-3.12) | **<0.001** |
| Disease duration, months | 1.00 (1.00-1.00) | 0.332 | 1.00 (1.00-1.00) | 0.905 |
| Comorbidities | 1.01 (0.81-1.27) | 0.924 | 0.96 (0.73-1.27) | 0.774 |
| Autoimmune comorbidities | 0.80 (0.53-1.22) | 0.301 | 0.89 (0.57-1.38) | 0.598 |
| Thymoma | 1.50 (1.18-1.89) | **0.001** | 1.94 (1.41-2.67) | **<0.001** |
| Thymectomy | 0.88 (0.69-1.14) | 0.330 | 0.58 (0.41-0.82) | **0.002** |
| Treatment |  |  |  |  |
| No therapy | 1 (Reference) |  | 1 (Reference) |  |
| Pyridostigmine only | 1.01 (0.75-1.35) | 0.958 | 0.98 (0.71-1.37) | 0.922 |
| Immunosuppressant | 0.90 (0.70-1.18) | 0.451 | 0.91 (0.68-1.23) | 0.546 |
| **Environmental characteristics** |  |  |  |  |
| Latitude |  |  |  |  |
| Low | 1 (Reference) |  | 1 (Reference) |  |
| Medium | 1.22 (0.96-1.56) | 0.111 | 1.66 (1.18-2.35) | **0.004** |
| High | 1.71 (1.20-2.43) | **0.003** | 1.48 (0.87-2.50) | 0.147 |
| Altitude |  |  |  |  |
| Low | 1 (Reference) |  | 1 (Reference) |  |
| Medium | 0.94 (0.71-1.23) | 0.635 | 0.69 (0.49-0.99) | **0.041** |
| High | 1.81 (1.29-2.54) | **0.001** | 1.56 (1.04-2.34) | **0.032** |
| Temperature |  |  |  |  |
| Low | 1 (Reference) |  | 1 (Reference) |  |
| Medium | 0.88 (0.69-1.11) | 0.286 | 1.20 (0.87-1.64) | 0.262 |
| High | 0.80 (0.63-1.03) | 0.084 | 1.01 (0.65-1.56) | 0.961 |
| Sunlight duration |  |  |  |  |
| Low | 1 (Reference) |  | 1 (Reference) |  |
| Medium | 0.64 (0.50-0.82) | **0.001** | 0.62 (0.46-0.86) | **0.004** |
| High | 1.08 (0.85-1.36) | 0.539 | 1.03 (0.70-1.51) | 0.880 |
| PM2.5 |  |  |  |  |
| Low | 1 (Reference) |  | 1 (Reference) |  |
| Medium | 0.76 (0.60-0.96) | **0.024** | 0.67 (0.50-0.91) | **0.011** |
| High | 0.70 (0.54-0.89) | **0.004** | 0.62 (0.43-0.89) | **0.009** |
| University education or above | 0.62 (0.46-0.83) | **0.001** | 0.72 (0.52-1.00) | **0.048** |
| PDI |  |  |  |  |
| Low | 1 (Reference) |  | 1 (Reference) |  |
| Medium | 0.78 (0.57-1.06) | 0.111 | 0.86 (0.61-1.21) | 0.395 |
| High | 0.58 (0.45-0.76) | **<0.001** | 0.60 (0.45-0.81) | **0.001** |
| Employed | 0.90 (0.73-1.11) | 0.312 | 1.15 (0.89-1.48) | 0.277 |
| Smoking | 0.96 (0.76-1.21) | 0.711 | 1.27 (0.89-1.82) | 0.184 |
| Alcohol consumption | 0.90 (0.70-1.16) | 0.418 | 1.20 (0.84-1.72) | 0.317 |
| Physical activity |  |  |  |  |
| Low | 1 (Reference) |  |  |  |
| Medium | 0.91 (0.70-1.17) | 0.455 | 0.96 (0.72-1.29) | 0.806 |
| High | 1.27 (0.82-1.97) | 0.279 | 1.20 (0.74-1.95) | 0.469 |
| Abbreviations: OR = odds ratio; CI = confidence interval; BMI = body mass index; PDI = per capita disposable income.  ^a^ ORs and 95% CIs were based on imputed dataset. Bold values are statistically significant (p < 0.05). | | | | |

**Fig. S1** Missingness map
